# Metabolic diseases and Kupffer cell’s plasticity

**DOI:** 10.1097/IN9.0000000000000066

**Published:** 2025-07-29

**Authors:** Francesca Fantini, Giuseppe Danilo Norata

**Affiliations:** 1 Department of Pharmacological and Biomolecular Sciences “Rodolfo Paoletti”, Università Degli Studi di Milano, Milan, Italy

**Keywords:** Kupffer cells, immunometabolism, cell plasticity

## Abstract

Macrophages play a crucial role in the innate immune system. They are present in most tissues, where they contribute to maintain homeostasis. Kupffer cells have specialized immunometabolic functions that link immune regulation and metabolic homeostasis directly. This enables them to regulate hepatic metabolism by controlling lipid handling and inflammatory responses. Consequently, there is growing interest in developing strategies to selectively modulate the function, polarity, distribution, behavior, and phenotype of Kupffer cells depending on the pathophysiological context. Given their plasticity and contribution to metabolic dysfunction-associated steatotic liver disease (MASLD), it is of increasing interest to find strategies that can selectively modulate Kupffer cell’s plasticity to control their distribution and phenotype depending on the pathophysiological context. This would modify their interaction with other cells in the liver niche, particularly hepatocytes, in the context of both atherosclerosis and MASLD. Future perspectives should focus on understanding how changes in the uptake capacity of Kupffer cells occur under conditions of lipid overload, and on exploring paracrine signals within the liver that can modulate their activation using advanced techniques such as high resolution spatial liver profiling.

Macrophages are crucial players in the innate immune system and are present in most tissues as a key component in maintaining homeostasis over a wide range of contexts in which they must both preserve the local environment and scavenge potentially harmful debris and pathogens by responding rapidly to specific stimuli ^[1]^. A number of recent studies contribute to define the characteristics of immune populations residing in the liver, allowing for a better distinction between antigen presenting cells such as Kupffer cells (KCs) and the other distinct subpopulations of liver macrophages under both homeostatic and nonhomeostatic conditions ^[2]^.

In homeostatic conditions, there are two main macrophage populations present in the liver: embryonic Kupffer Cells (EmKC) and capsular macrophages ^[3]^. KCs are the resident macrophages in the liver and represent one of the most abundant macrophage populations in the entire organism (80%–90% of tissue macrophages) ^[4]^. This cluster of cells is defined by the expression of specific markers such as Clec4F, Tim4, Visig4, and Marco in addition to common macrophage markers such as F4/80 and CD64 and differs from capsular macrophages not only for their localization but also because they lack Cx3Cr1 expression ^[1,2]^. KCs reside in the hepatic niche and are distributed throughout the functional unit of the hepatic lobule with an increasing gradient of concentration toward the portal vein, from which they can easily capture exogenous material coming from the gastrointestinal tract via the portal vein and subsequently make contact with surrounding hepatic cells to catabolize the received content. Of note, KCs are not strictly localized within blood vessels but always extend a major fraction of their cell body into the perisinusoidal Space of Disse, where they interact closely with liver sinusoidal endothelial cells, hepatic stellate cells, and hepatocytes. The localization of KCs appears to be crucial for their activity, including the capability to uptake aged red blood cells from blood vessels and transfer iron to hepatocytes ^[5]^ (Figure 1).

KCs are long-lived self-renewing macrophages; however, when they undergo depletion, such as in the presence of a lipid enriched environment, circulating monocytes, which usually pervade liver vessels but do not contribute to the macrophage pool, are rapidly recruited and settle in the liver gradually adopting the transcriptional profile of their depleted counterparts. After a time window of nearly two weeks, they become long-lived self-renewing cells and acquire the ability to self-maintain in the liver without any subsequent contribution from bone marrow-derived monocytes. Thus, like the embryonic precursors, the monocytes themselves can give rise to tissue-resident self-renewing macrophages when the hepatic niche is available to receive and educate them, that is, when KCs’ death allows engraftment of the monocytes themselves, and the surrounding cells provide for their education ^[6]^. Macrophages derived from bone marrow have, thus, a different origin compared with those residing in tissues of embryonic origin, yet they reach a genetic signature comparable to that of resident macrophages in about seven days. This process questions the dogma of macrophage ontogeny and supports the hypothesis that the amount of time a macrophage spends in a particular tissue environment or niche could be responsible for its signature as a consequence of the constant instructions received from other cells in that niche ^[1,7,8]^. Initial in vitro experiments have shown that EmKCs differ from monocytic KCs (MoKCs) in their response to stimulation, yet a number of in vivo studies have shown that bone marrow-derived macrophages are capable of giving rise to self-renewing and fully differentiated KCs ^[6,8]^. The field is still controversial, but an increasing number of findings support the hypothesis that MoKCs can acquire EmKC-like characteristics, including the ability to self-renew as well as morphological and gene expression profile similarities ^[6]^. However, despite their origins, the specific functional differences that distinct subpopulations of KCs may acquire within pathological contexts contribute differently to the maintenance of hepatic homeostasis as well as to the pathological progression ^[9,10]^ (Figure 1).

The mechanisms underlying the differentiation and function of KCs and macrophages in metabolic dysfunction-associated steatotic liver disease (MASLD) and/or steatohepatitis are still under intensive investigation with the acquisition of distinct pro- or anti-inflammatory patterns able to influence the severity and progression of liver disease. Recently, significant progress has been made in elucidating the temporal and spatial dynamics of infiltrating monocytes and monocyte-derived macrophage subpopulations in MASLD using single-cell RNA sequencing and fate mapping of hepatic macrophage subpopulations. The Notch-RBPJ signaling pathway appears to be crucial in controlling the monocyte-macrophage transition. RBPJ deficiency blocks inflammatory macrophages and monocyte-derived KC differentiation while promoting the emergence of protective Ly6C low monocytes. Mechanistically, RBPJ deficiency promotes lipid uptake in Ly6C low monocytes, driven by elevated CD36 expression, thereby enhancing their protective interactions with endothelial cells ^[11]^. Regardless of their origin, KCs have been shown to play a key role in several experimental settings. Beyond efferocytosis of pathogens or debris, they can recognize and trap ApoB-containing lipoproteins, which are well known to play a critical pathological role in the development of atherosclerosis and are correlated with the risk of MASLD development. This new evidence strengthens the link between cholesterol-rich ApoB-containing lipoproteins, the immune system, and liver inflammation. KCs participate in the systemic response associated with atherogenesis through their early response to an atherogenic diet. In particular, EmKCs undergo massive expansion immediately after induction of hypercholesterolemia, which is advantageous because they are able to accumulate large amounts of lipoprotein-derived cholesterol, in part via the CD36 scavenger receptor, which has been implicated in atherogenesis through its function in recognizing modified endogenous ligands such as oxLDL. After this rapid adaptive response, as hypercholesterolemia progresses, the already cholesterol-loaded EmKCs are subjected to increased mitochondrial oxidative stress and endoplasmic reticulum damage, leading to apoptosis, and their number gradually decreases, while MoKCs, with a lower capacity to load cholesterol, are recruited from the circulation to supplement the KC pool. The decrease in the proportion of MoKCs in the KC pool increases the cholesterol content in the liver and aggravates hypercholesterolemia, leading to accelerated atherosclerotic plaque development, confirming how overall KC homeostasis is disrupted in hypercholesterolemia, altering plasma cholesterol control, and increasing atherosclerosis. Notably, depletion of KCs with clodronate abolished the hepatic transcriptional response to acute dyslipidemia, confirming the close link between ApoB-containing particles, the immune system, and liver metabolism ^[12]^. Moreover, the role of CD36 was also confirmed by examining different types of lipid-associated macrophages in multiple models of liver injury, where its expression is necessary for the efficient elimination of dying cells to enhance repair and prevent exacerbated fibrosis ^[13]^.

Recent studies have also confirmed that in a model of MASLD, KCs can take up oxidized LDL and accumulate lysosomal free cholesterol, leading to an inflammatory response. By removing ApoB-containing lipoproteins and releasing anti-inflammatory mediators, KCs tend to reduce the circulation of atherogenic lipids, thereby protecting against atherosclerosis. Conversely, KCs can also play a detrimental role in atherosclerosis by promoting the release of pro-inflammatory mediators. This evidence suggests that the dual behavior of KCs may depend on their plasticity in response to changes in the microenvironment ^[12,14]^.

In fact, two functionally distinct populations of KCs have been identified, termed KC1 and KC2, which can be analogized to the similar nomenclature used for macrophages to identify the former as more prone to inflammatory phenotypes and the latter as proresolving, considering the many shades between these two polarized and simplistically opposed phenotypes. As a matter of fact, KCs can differentiate into two main types: M1-like (classical) and M2-like (alternative) macrophages, each with distinct functions and characteristics ^[15]^. M1-like KCs are induced by pathogen-associated molecular patterns and pro-inflammatory cytokines, and their main function is to produce pro-inflammatory mediators and contribute to inflammation and the generation of reactive oxygen species, which also contribute significantly to several liver pathologies such as MASLD ^[15,16]^. On the other hand, Type 2 KCs are more likely to be activated by anti-inflammatory signals or tissue repair processes, are able to produce anti-inflammatory mediators such as IL10, and play a role in liver remodeling and fibrosis resolution in favor of homeostasis ^[16]^.

In recent years, the KC2 subset of embryonic origin has been highlighted as an interesting cellular subpopulation, characterized by specific markers (CD206highESAM+) and showing a metabolic function linked to the upregulation of pivotal genes involved in carbohydrate and lipid metabolism. In fact, it has been shown that during diet-induced obesity, genes involved in fatty acid processing are upregulated in the KC2 subset, with specific reference to CD36, known for its role in lipid uptake and modulation of oxidative stress in macrophages. Furthermore, when compared to wild-type controls on a high-fat diet, specific suppression of this KC2 subpopulation resulted in no weight gain, reduced oxidative stress and lipid peroxidation, improved glucose tolerance, and decreased steatosis. This suggests that this particular subpopulation could play a significant role in liver pathophysiology, particularly in the development of diet-induced obesity ^[17]^.

Taken together, the complexity of this information suggests that KCs have specialized immunometabolic functions that directly link immune regulation and metabolic homeostasis; in fact, these cells have unique transcriptional and functional profiles compared with other macrophages in body districts, and this allows them to modulate hepatic metabolism through lipid handling, but also inflammatory responses through paracrine signaling about which little is yet known ^[18]^ (Figure 1). It is, therefore, of growing interest to find strategies to selectively modulate the function and polarity of KCs, both to potentially control their distribution and behavior, and to be able to modulate their phenotype depending on the pathophysiological context, to modify their cross-talk with other cells in the liver niche, in particular hepatocytes in the context of atherosclerosis and MASLD.

Future research should aim to fill some interesting gaps related to KC biology, such as (i) unraveling the mechanism underlying the loss of lipid uptake capacity of EmKCs under conditions of lipid overload and how this might affect hypercholesterolemia dependent generation of dysfunctional KCs; (ii) understand the paracrine signals within the hepatic niche that may activate stellate cells, thus leading to the increase in fibrosis and exacerbate MASLD progression. To achieve these ambitious aims, in addition to in vivo and in vitro studies investigating the interactions between KCs and other cells in the hepatic niche, spatial profiling of the liver and high-resolution bioinformatics analysis are potentially key tools for gaining a better understanding of whether there are functional differences between KCs relating to their localization along the central vein–portal vein axis, and how this affects their cross-talk with hepatocytes.

## Figures and Tables

**Figure 1:**
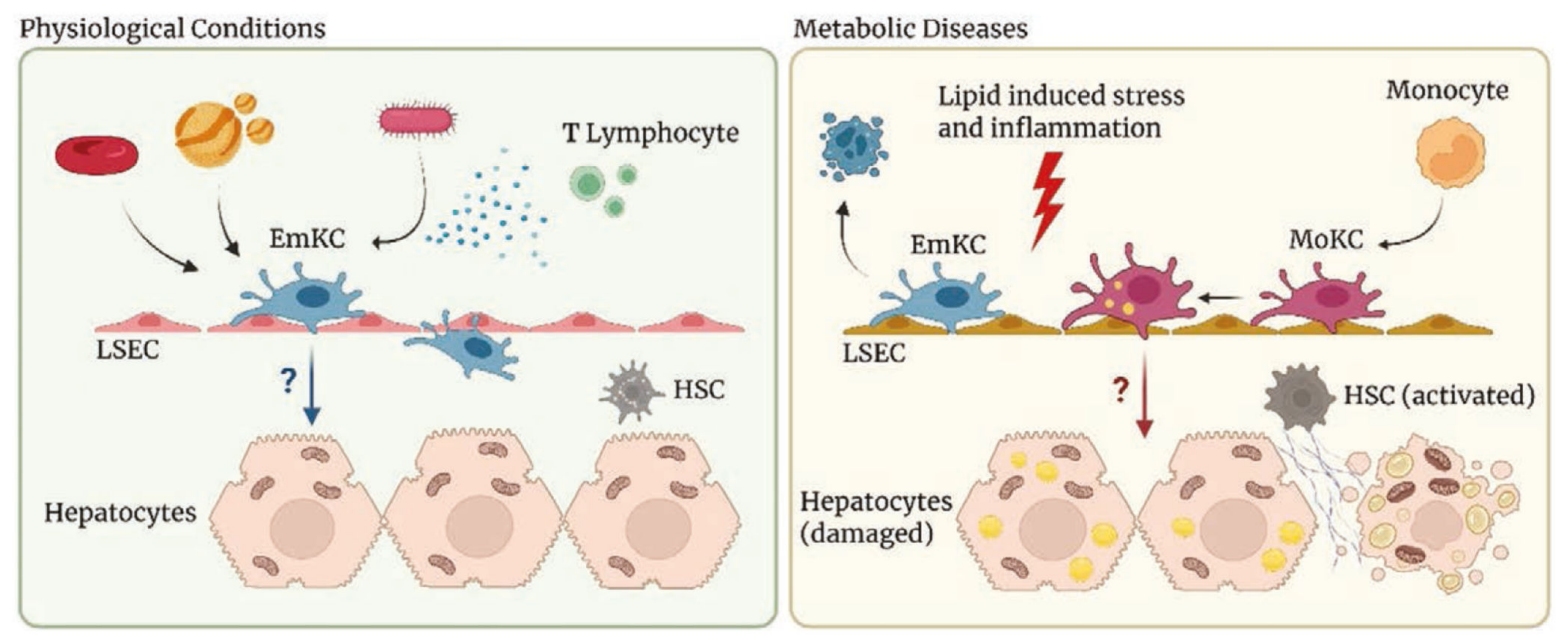
In homeostatic conditions (left), Kupffer cells contribute to maintain liver homeostasis by scavenging aged red blood cells, lipoproteins, and potentially harmful pathogens. Their unique location facilitates close interaction with liver sinusoidal endothelial cells (LSECs), hepatic stellate cells (HSCs), and hepatocytes, resulting in a rapid response to distinct stimuli. In the context of metabolic diseases (right), both lipid-induced stress and inflammation lead to the depletion of Kupffer cells. Monocytes are rapidly recruited to the liver, where they differentiate into cells identical to Kupffer cells. In both contexts, the molecular mechanisms that control the functional modulation of Kupffer cells and the crosstalk with the hepatic niche are not yet fully understood.
